# Pathway aberrations of murine melanoma cells observed in Paired-End diTag transcriptomes

**DOI:** 10.1186/1471-2407-7-109

**Published:** 2007-06-26

**Authors:** Kuo Ping Chiu, Pramila Ariyaratne, Han Xu, Adrian Tan, Patrick Ng, Edison Tak-Bun Liu, Yijun Ruan, Chia-Lin Wei, Wing-Kin Ken Sung

**Affiliations:** 1 Genome Institute of Singapore, 60 Biopolis Street, Genome #02-01, 138672, Singapore; 2 Department of Computer Science, National University of Singapore, 3 Science Drive 2, 117543, Singapore

## Abstract

**Background:**

Melanoma is the major cause of skin cancer deaths and melanoma incidence doubles every 10 to 20 years. However, little is known about melanoma pathway aberrations. Here we applied the robust Gene Identification Signature Paired End diTag (GIS-PET) approach to investigate the melanoma transcriptome and characterize the global pathway aberrations.

**Methods:**

GIS-PET technology directly links 5' mRNA signatures with their corresponding 3' signatures to generate, and then concatenate, PETs for efficient sequencing. We annotated PETs to pathways of KEGG database and compared the murine B16F1 melanoma transcriptome with three non-melanoma murine transcriptomes (Melan-a2 melanocytes, E14 embryonic stem cells, and E17.5 embryo). Gene expression levels as represented by PET counts were compared across melanoma and melanocyte libraries to identify the most significantly altered pathways and investigate the expression levels of crucial cancer genes.

**Results:**

Melanin biosynthesis genes were solely expressed in the cells of melanocytic origin, indicating the feasibility of using the PET approach for transcriptome comparison. The most significantly altered pathways were metabolic pathways, including upregulated pathways: purine metabolism, aminophosphonate metabolism, tyrosine metabolism, selenoamino acid metabolism, galactose utilization, nitrobenzene degradation, and bisphenol A degradation; and downregulated pathways: oxidative phosphorylation, ATPase synthesis, TCA cycle, pyruvate metabolism, and glutathione metabolism. The downregulated pathways concurrently indicated a slowdown of mitochondrial activities. Mitochondrial permeability was also significantly altered, as indicated by transcriptional activation of ATP/ADP, citrate/malate, Mg^++^, fatty acid and amino acid transporters, and transcriptional repression of zinc and metal ion transporters. Upregulation of cell cycle progression, MAPK, and PI3K/Akt pathways were more limited to certain region(s) of the pathway. Expression levels of c-*Myc *and *Trp53 *were also higher in melanoma. Moreover, transcriptional variants resulted from alternative transcription start sites or alternative polyadenylation sites were found in *Ras *and genes encoding adhesion or cytoskeleton proteins such as integrin, β-catenin, α-catenin, and actin.

**Conclusion:**

The highly correlated results unmistakably point to a systematic downregulation of mitochondrial activities, which we hypothesize aims to downgrade the mitochondria-mediated apoptosis and the dependency of cancer cells on angiogenesis. Our results also demonstrate the advantage of using the PET approach in conjunction with KEGG database for systematic pathway analysis.

## Background

Cancers are caused by multiple genetic and/or epigenetic alterations [1-4]. These alterations include activation of oncogenes, inactivation of tumor suppressor genes, mutations that cause chromosome instability [5], and mutations that affect key pathways such as apoptosis, MAPK, cell cycle progression, Wnt/β-catenin, metastasis, and angiogenesis [6-9].

Melanomas are among the most common cancers in human and their incidences continue to rise at a pace faster than any other malignancy [10]. Genetic alterations in melanoma signaling pathways have been reported recently [3,11]; however, global pathway aberrations remain unclear. We applied the robust Gene Identification Signature Paired-End diTag technology (GIS-PET) to reveal the global pathway aberrations in melanoma by using the murine melanoma cell line B16F1 as a model system. B16F1 is a metastatic clone generated from the spontaneous melanoma cell line B16F0. Some *in vitro *and *in vivo *studies of this cell line, including deletion in *Ink4a*/*Arf *exons and p53 protein expression level, have been well documented and can serve as controls for data validation [12,13].

Previous transcriptome studies were mostly performed with high throughput microarray or Serial Analysis of Gene Expression (SAGE) approaches. Microarray is a well commercialized technology [14]. It uses mRNAs from a given cell line or tissue to generate a labeled target sample, which is hybridized to a large number of DNA sequences, each representing a gene. The signal intensity of each hybridized DNA sequence is subtracted by a control and analyzed with software packages not only for data processing, but also for mapping gene-expression clusters to integrated pathways [15]. SAGE is another powerful method for studying transcriptome profiles. It extracts short, positionally defined, tag signatures from expressed mRNAs and subsequently correlates the signatures to genomic coordinates using the UniGene virtual database [16,17]. The SAGE method is also supported by a number of software and public databases which have been made available for cancer studies [18,19]. Both of these approaches have been applied to melanoma studies. The focuses of these studies, however, were mainly on genes, gene sets, or pathway annotations. To our best knowledge, application of these technologies (or any other technologies) to the global study of melanoma pathway aberrations is presently not available.

GIS-PET was originally developed to facilitate the study of transcriptome profiles. It covalently links the corresponding 5' and 3' signatures of full-length transcripts into PET sequences of around 36 bp (18 bp from each of the 5' and 3' signatures of the same mRNA transcript) and concatenates multiple PETs to form longer stretches of cDNA fragments for high throughput sequencing [20,21]. We have shown that GIS-PET is able to precisely locate the transcription start sites (TSSs) and the polyadenylation sites (PASs) of expressed genes. When combined with chromatin immunoprecipitation (ChIP) approach, PET method is able to precisely map the genome-wide transcription factor binding sites (TFBSs), as demonstrated in the studies of human tumor suppressor p53 [22] and murine developmental regulators OCT4 and NANOG [23].

For this investigation, we generated PET transcriptomes from 4 independent but related murine cell types: B16F1 melanoma cells, Melan-a2 melanocytes, E17.5 embryo, and E14 embryonic stem cells. PETs were mapped against University of California at Santa Cruz (UCSC) genome database mm5 and annotated to known genes. The associated genes were subsequently annotated to various pathways using KEGG murine database. We adopted hypergeometric distribution to assess pathway alterations based on a few reasons. First, hypergeometric distribution is one of the most commonly used analytical methods for microarray transcriptome analysis. Although there are differences between PET and microarray technologies, their analytical tools are mutually applicable to a great extent. Secondly, our transcriptome libraries contain large datasets, and variations resulted from the differences in analytical metrics become insignificant for large datasets. Moreover, our libraries do not contain replicates or serial samples collected from different time points and are thus suitable for analysis with the hypergeometric approach.

Because melanocytic cells (including B16F1 and Melan-a2) have the unique capability to produce melanin, while embryonic cells (including E14 stem cells and E17.5 embryo) do not, we first compared the PET counts of melanin biosynthesis genes between melanocytic cells and embryonic cells to ensure the feasibility of using PET count to represent the expression level. Melanogenesis in mice is a complex process involving more than 150 genes located in about 50 loci, which exert functions in various processes including melanin biosynthesis, aggregation and transportation [24] We focused on melanin biosynthesis because this process has been well characterized. The results strongly support the idea of using a PET approach for pathway comparisons.

We subsequently evaluate the degree of pathway perturbation library-wide using the hypergeometric approach so as to identify the most significantly altered pathways in melanoma cells. We also looked into the pathways previously shown to be implicated in tumorigenesis and identified the transcriptional variants resulted from alternative TSSs or alternative PASs, which may play important roles in melanoma tumorigenesis and pathway aberrations. Our data revealed a systematic redistribution of workload for multiple metabolic pathways leading to an enhanced detoxification capability and a reduction of electron transport chain (ETC) usage through a global downregulation of mitochondria-harbored pathways. In parallel, the revamping was accompanied by shattered tumor suppressor pathways and apoptotic pathways, and redefined mitochondrial permeability. Thus, functional perturbations in multiple molecules, caused by multiple genetic and/or epigenetic alterations, affect multiple metabolic and signal transduction pathways and the combinatorial effect ultimately determines cancer cell survival. Our discoveries should be able to facilitate drug target discoveries for cancer therapy.

## Methods

### Cell cultures

As described previously [25], B16F1 murine melanoma cells were grown in Dulbecco's modified Eagle's medium (DMEM) supplemented with 10% fetal bovine serum (FBS, Gibco-BRL), plus penicillin (100 u/ml), and streptomycin (100 μg/ml, Invitrogen). Cells were cultured at 37°C with 5% CO_2_. Melan-a2 murine melanocytes were grown in RPMI 1640 medium supplemented with 10% FBS (Gibco-BRL), plus penicillin (100 u/ml), streptomycin (100 μg/ml), 12-0-tetradecanoyl phorbol acetate (200 nM, Sigma), and cholera toxin (200 pM, Sigma). Cells were cultured at 37°C with 10% CO_2_. Embryonic day 14 (E14) mouse ES cells were either co-cultured with mouse primary embryonic fibroblast feeders or cultured under feeder-free conditions [23]. Cells were maintained in DMEM (GIBCO), supplemented with 15% heat-inactivated fetal bovine serum (GIBCO), plus β-mercaptoethanol (0.055 mM, GIBCO), L-glutamine (2 mM), MEM (0.1 mM nonessential amino acid), penicillin/streptomycin (5,000 units/ml) and leukemia inhibitory factor (1,000 units/ml, Chemicon).

### Library construction and PET extraction

Methods for constructing GIS-PET transcriptome libraries and scripts for PET extraction are available for download [26], and can also be found from our previous publications [20,27]. Spacers, which interpose PETs in the raw sequences, are defined by the vectors and restriction enzymes used for library constructions. The 5' most spacer is denoted as 5' spacer; the 3' most spacer as 3' spacer; the major internal spacer as spacer1; and the minor internal spacer, which might be generated from incomplete enzymatic manipulation, as spacer2. Libraries SMT001 (B16F1 melanoma library), SMN001 (Melan-a2 melanocyte library) and SME006 (E17.5 embryo library) had the same 5' spacer (GATCGAC), spacer1 (GTCGGATCCGAC), spacer2 (GTCGCGAC), and 3' spacer (GTCGATC), while library MoEScom (E14 embryonic stem cell library) had all spacers of the same sequence 'GTCGGATCCGAC'. The minimum and maximum ditag lengths were 34 and 40, respectively. PET-Tool first identified the spacers and extracted the PET candidates interspersed between the spacers. PET candidates containing more than 8 bp of consecutive A, T, G, or C in either the 5' or 3' tag region were removed, due to the facts that these PET sequences were very likely to be artifacts or contaminants introduced in the wet-lab. N-containing PETs were also removed. The PET extraction process generated a pool of unique PETs (each with a unique sequence) for each library, and each unique PET might have a single or multiple copy number(s), termed PET count. Prior to library comparison, PET counts were normalized to counts per million (cpm) based on the total PET counts of the original libraries, i.e. Every PET count was scaled up by the factor 1,000,000/(total PET count) before the comparison.

### PET-to-genome mapping

Compressed Suffix Arrays (CSA) constructed from the UCSC mouse genome assembly mm5 were used for mapping. Each PET was split into a 5' tag and a 3' tag. From the 5' tag, a set of 9 subsequences were generated by allowing the tag subsequence to start from position 1, 2, or 3, and end at position 17, 18, or 19. The remaining portions formed the 3' subsequences. These subsequences were subjected to mapping against the CSA chromosome constructs. The mapping process identified the chromosome location(s), if present, for each subsequence. Mismatches, deletions, and insertions were not allowed during mapping and the minimum perfect match lengths for the 5' and 3' subsequences were set to 16 and 14 bp, respectively. The 5' hits were then paired with the 3' hits to identify the genomic target locations of the PETs. The criteria for pairing were: both the 5' and 3' mapping locations had to be on the same chromosome, same strand, in 5' followed by 3' order, and within 1 million bp in distance. PETs were then split into PET1 - PET>10 categories based on the number of genomic targets. PETs that could not be mapped under these parameters were assigned to the PET0 category; PETs that mapped to single locations were assigned to the PET1 category; and so on. This study used only PET1 ditags to avoid complication. PET sequence data will be available to the public through T2G website [28] when the paper is published.

### Individual PET-to-exon designation

**Figure 1:**
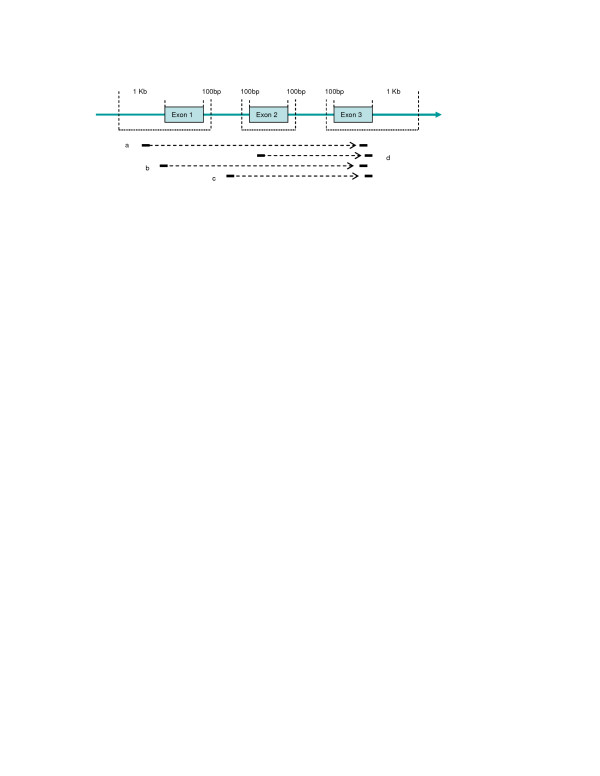
**PET-to-gene annotation**. PET-to-gene annotation is based on tag-to-exon annotation, through which the mapping locations of the 5' and 3' tags of each PET1 ditags (PETs with unique targets) are compared with exon locations of the knownGene, refSeq and MGC genes of UCSC mm5 database. To absorb the variations that may be present, exon boundaries are extended 100 bp (for internal boundaries) or 1 Kb (for external boundaries). Under such definition, both 'a' and 'b' (5' tags) match to exon 1, and both transcripts are denoted as S1E3T3; 'c' (5' tag) matches to intron 1, and the transcript is denoted as S1-2E3T3; while 'd' (3' tag) matches to exon 3, and the transcript is denoted as S2E3T3. All these PETs are annotated to the same gene. S, starting exon; E, ending exon; T, total exon.

To assign PET tags to exons, we compared the 5' and 3' mapping locations of each PET to the known gene exons downloaded from the knownGene, refSeq, and MGC tables of the UCSC annotation database (herein, a known gene refers to a gene that matches any of these sources). The PET-to-exon designation is the basis for the "PET-to-transcription unit" annotation. For a PET generated from an mRNA transcribed from a known gene, the 5' tag is expected to map to the first exon and the 3' tag to the last exon of the same gene. However, there were situations where a PET mapped to a novel exon of a known gene or it might map to a known exon but with an offset. To absorb variations in exon boundaries, we extended the starting and ending positions of each exon outwards by 100 bp (Figure 1). A tag, either the 5' or the 3' tag, with a mapping location within such defined exon boundaries was considered a successful match; otherwise, if either end of a PET mapped to the intronic region, it was considered as an intragenic tag. For the terminal exons, we extended the range by 1 Kb, to include plausible novel TSSs or PASs.

If only one side, either the 5' or the 3' tag, matched an exon of a known gene, more stringent criteria were applied to avoid faulty exon annotation. The criteria were: the span between the 5' and 3' tags had to be less than 300,000 bp, and the minimum perfect match lengths for the 5' tag and the 3' tag had to be at least 17 bp and 15 bp, respectively, instead of 16 bp and 14 bp used in default mapping.

We also used a 'SET' system to represent the qualities and natures of PETs that mapped to a particular "transcription unit" ('S', 'E' and 'T' stands for the starting exon, ending exon, and total number of exons, respectively). As an example of a perfect PET-to-transcription unit annotation, 'S1E5T5' indicates that the 5' tag matches to exon1, and the 3' tag matches to exon5 of the same transcription unit that has a total of 5 exons. As an example of an imperfect PET-to-transcription unit annotation, S1E3-4T5 indicates that the 5' tag matches to exon1, and the 3' tag matches to the third intron of the same transcription unit that has a total of 5 exons. The geneID of the transcription unit that owned the PET-associated exon(s) was annotated to the PET with the expression level shown as the PET count. For UCSC database, each geneID has a corresponding geneSymbol (terminology as in UCSC annotation database) and multiple geneIDs may point to the same geneSymbol (N:1 relation).

### PET clustering and cluster-to-gene annotation

Here a GIS-PET cluster is defined as a genomic origin, or a gene, that expresses a group of related transcription units. In practice, PET clustering was conducted for two categories separately: one for known gene annotated PETs (gene-based) and the other for un-annotated PETs (coordinate-based, or chromosome location-based). The gene-based clustering was conducted by the following steps: First, all PETs were separated based on the 'SET' designation. PETs with the same starting and ending exons were grouped into the same "sub-cluster" representing a distinguishable "transcription unit", and their counts were summed to represent the expression level of that transcription unit. Second, all the transcripts belonging to the same geneSymbol were further grouped into the same "cluster" with PET counts summed to represent the overall expression level of the gene. Lastly, for a cluster containing multiple geneSymbols, the geneSymbol associated with the majority of PETs was annotated to that cluster and all PET counts were summed to represent the expression level of that gene cluster. Since only known gene PETs can be correlated to the KEGG pathways, there will be no further discussion of PETs clustered by the coordinate-based clustering approach.

### Gene level melanoma-melanocyte comparison

**Figure 2:**
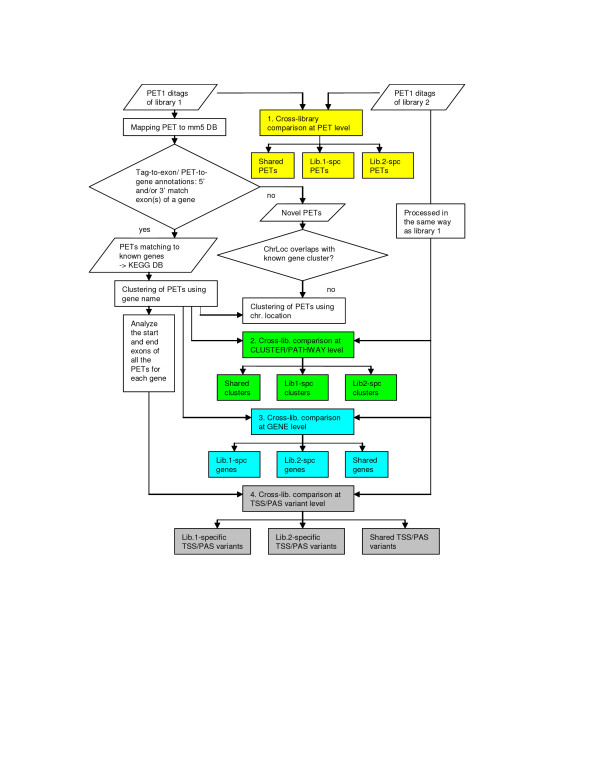
**PET clustering and library comparison**. PETs are clustered first based on their annotated known genes, if available. PETs that cannot be annotated to any known gene are clustered based on their chromosome locations (genome coordinates). Library comparisons can be conducted at various levels including PET, cluster, gene, and TSS/PAS variant. The PET level comparison measures the degree of overlap between unique PETs across two datasets. The cluster level comparison evaluates the similarity between two libraries for both known gene clusters and novel clusters, while the gene level comparison considers only known gene clusters. The TSS/PAS variant comparison compares alternative TSSs and PASs present in the known gene clusters. TSS, transcription start site; PAS, polyadenylation site; chrLoc, chromosome location.

B16F1 melanoma and Melan-a2 melanocyte transcriptomes were compared using known genes present in either or both libraries (Figure 2). Results were partitioned into 3 mutually exclusive sets: genes that are exclusive to the B16F1 melanoma library, genes that are shared by both libraries, and genes that are exclusive to the Melan-a2 library. Genes highly expressed in melanoma, but not in melanocytes (or with low expression levels) were listed.

### Pathway level melanoma-melanocyte comparison

**Figure 3:**
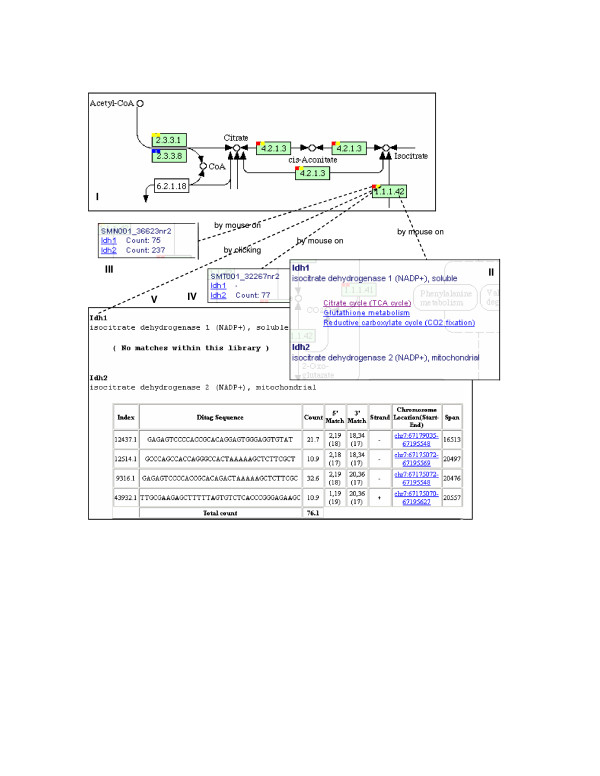
**PET-to-pathway display**. The KEGG pathway display (I) was modified to reflect library information. Solid squares were added to the upper left corner of the gene box (green icon, with 4 numbers to represent a particular KEGG gene ID). Each square is associated with a library and highlighted with red, yellow, or blue color to indicate that gene isoforms are all matched, partially matched, or completely unmatched, respectively, by PETs of the library. By placing the cursor on a particular entity (mouse on), one can view the description of all gene isoforms in the gene box (II), or PET counts, in cpm (counts per million), of a corresponding gene isoform for each library (III & IV). In response to a clicking on a solid square, detailed mapping information is displayed (V). Hyperlinks in II lead to all KEGG pathway images that contain the selected gene isoform.

**Figure 4:**
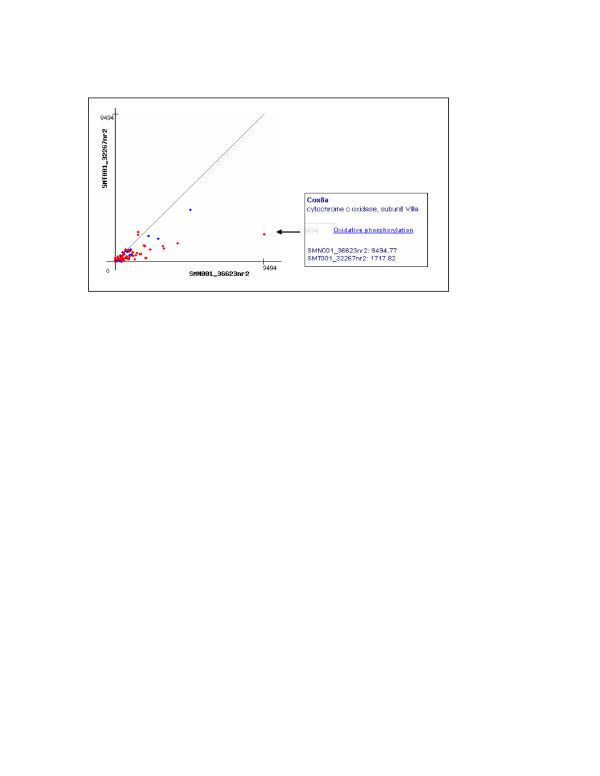
**Expression graph of oxidative phosphorylation and ATP synthesis pathways**. PET counts, in cpm (counts per million), of gene isoforms in the oxidative phosphorylation pathway (red) and the ATP synthesis pathway (blue) are plotted to show that both pathways are downregulated in B16F1 melanoma cells (library SMT001_32267nr2), compared with Melan-a2 melanocytes (SMN001_36623nr2). This function is implemented in a local KEGG-PET application for pathway analysis. As exemplified by the box in the lower-right corner, PET counts of each gene isoform (*Cox8a *in this case) for all libraries are accessible by using mouse-on method.

To facilitate data analysis, we built a local mirror site of KEGG pathway, containing a total of 141 pathways, with a slight modification to reflect our local data status (Figure 3). PET associated genes were correlated to their corresponding KEGG IDs using the file 'keggPathway.txt' available at UCSC database. If the knownGene ID for a PET cluster was not available (e.g. the PETs mapped to a refGene entity) we either converted refGene ID to knownGene ID using 'kgXref.txt' or used the gene symbol to map it to the corresponding KEGG ID. We modified the original pathway images by adding solid squares, each representing a library, to the upper left corner of the gene box to indicate the status of isoform coverage by the libraries. The solid squares were highlighted red, yellow, or blue to indicate that the isoforms all matched, partially matched, or did not match at all, respectively. Links were also created on the solid boxes to allow easy access to additional information. Multiple PET libraries could be displayed in the same page to facilitate side-by-side comparison. Another feature added to the local KEGG pathway database was the expression graph for making cross library comparison over PET counts of all gene isoforms of a particular pathway to enhance visualization (Figure 4).

### Testing the feasibility of using PET count to measure gene expression level

The melanin biosynthesis pathway was not listed in the KEGG database, and the analysis was conducted alternatively with the following steps: First, we used UCSC mouse genome database mm5 and the Gene Ontology database [29] to identify all the melanin biosynthesis related genes. Second, PETs mapped to these genes were identified from all four libraries and their counts were added up for each gene and each library. Then, the PET counts of each gene were compared across all four libraries.

### Identification of most significantly altered pathways with hypergeometric distribution

Global pathway comparison was conducted by comparing all pathways using a hypergeometric distribution method with a minor modification to identify the pathways with most significant aberrations. This was conducted by the following steps: Given a gene, its expression ratio *r *was defined as:

r=(number of PET counts in melanoma)+s0(number of PET counts in melanocyte)+s0
 MathType@MTEF@5@5@+=feaafiart1ev1aaatCvAUfKttLearuWrP9MDH5MBPbIqV92AaeXatLxBI9gBaebbnrfifHhDYfgasaacH8akY=wiFfYdH8Gipec8Eeeu0xXdbba9frFj0=OqFfea0dXdd9vqai=hGuQ8kuc9pgc9s8qqaq=dirpe0xb9q8qiLsFr0=vr0=vr0dc8meaabaqaciaacaGaaeqabaqabeGadaaakeaacqWGYbGCcqGH9aqpdaWcaaqaaiabcIcaOiabb6gaUjabbwha1jabb2gaTjabbkgaIjabbwgaLjabbkhaYjabbccaGiabb+gaVjabbAgaMjabbccaGiabbcfaqjabbweafjabbsfaujabbccaGiabbogaJjabb+gaVjabbwha1jabb6gaUjabbsha0jabbohaZjabbccaGiabbMgaPjabb6gaUjabbccaGiabb2gaTjabbwgaLjabbYgaSjabbggaHjabb6gaUjabb+gaVjabb2gaTjabbggaHjabcMcaPiabgUcaRiabdohaZnaaBaaaleaacqaIWaamaeqaaaGcbaGaeiikaGIaeeOBa4MaeeyDauNaeeyBa0MaeeOyaiMaeeyzauMaeeOCaiNaeeiiaaIaee4Ba8MaeeOzayMaeeiiaaIaeeiuaaLaeeyrauKaeeivaqLaeeiiaaIaee4yamMaee4Ba8MaeeyDauNaeeOBa4MaeeiDaqNaee4CamNaeeiiaaIaeeyAaKMaeeOBa4MaeeiiaaIaeeyBa0MaeeyzauMaeeiBaWMaeeyyaeMaeeOBa4Maee4Ba8Maee4yamMaeeyEaKNaeeiDaqNaeeyzauMaeiykaKIaey4kaSIaem4Cam3aaSbaaSqaaiabicdaWaqabaaaaaaa@8C9F@

where s_0 _is a small positive value representing the pseudocount for stabilizing the ratio when the expression level of the gene is low [30]. The s_0 _was empirically determined to be 5 in our study. With the criterion of 1.5-fold change as the cutoff, we identified upregulated genes (r ≥ 1.5), and downregulated genes (r ≤ 0.67). For each KEGG pathway, we calculated the hypergeometric p-value which indicates the enrichment of regulated genes in the pathway. Pathways with p-values of 0.005 or less were considered to be significantly altered. Manual curation was applied to ensure the quality of pathway analysis.

### TSS/PAS variants

We used the 'SET' transcription unit definition described above to differentiate transcriptional variants resulted from alternative TSSs or alternative PASs Transcription units sharing the same values of 'S', 'E' and 'T' were considered as common ones. Otherwise they were considered as library specific. The results were split into seven categories.

### Partial pathway analysis and manual curation

In general, KEGG pathways are loosely defined and some are partially overlapped with each other; thus, whole pathway comparison can be overgeneralized and overlooks local transcriptional architectures. To compensate this potential problem, some pathways were partially analyzed or manually curated (see Results and Discussion). For example, the galactose-glucose interconversion pathway was extracted from the galactose metabolism pathway to focus on genes directly involved in the galactose and glucose interconversion process. Similarly, the RAS-ERK sub-pathway was extracted from the MAPK pathway and the expression of cyclins and CDKs was obtained from the cell cycle pathway. Manual curation was conducted for the most significantly altered pathways to ensure accuracy.

## Results and discussion

### Libraries

The numbers of transcriptome PETs (including the duplicates) totaled 91,977 (equivalent to 45,465 unique PETs) and 68,880 (equivalent to 51,352 unique PETs) for the B16F1 melanoma library and Melan-a2 melanocyte library, respectively (Table 1). After mapping and further characterization, we identified 32,267 PET1 ditags from the melanoma transcriptome. Among these, 29,439 (91.2%) were associated with known genes, and 11,687 (36.2%) of the known gene-associated PET1 ditags were further annotated to KEGG pathways. The melanocyte transcriptome contained 36,623 PET1 ditags, 33,005 (90.1%) were associated with known genes, of which 13,348 (36.4%) were further annotated to KEGG pathways. Overall, the melanocyte library scored the highest success rate (40.4%) for known gene-to-pathway association, followed by the melanoma library (39.7%), the E17.5 day old embryo library (37.8%), and the E14 stem cell library (32.9%). The distinct difference in annotatable rate between the melanocytic libraries and the embryonic libraries (especially the E14 stem cell) suggests that certain developmental pathways are presently uncharacterized and not available in the KEGG databases.

**Table 1:** Library statistics.

Library	#Total PETs	#uPETs	#Mappable (% over uPETs)	# PET1	#Known gene-asso. PET1 (% over PET1)	#Pathway-asso. PET1 (% over PET1) (% over known gene-asso. PET1)
B16F1 melanoma (SMT001)	91977	45465	34146 (75.1%)	32267	29439 (91.2%)	11687 (36.2%) (39.7%)
Melan-a2 melanocyte (SMN001)	68880	51352	38640 (75.3%)	36623	33005 (90.1%)	13348 (36.4%) (40.4%)
E14 Stem Cell (MoEScom)	248234	135328	97295 (71.9%)	93384	86019 (92.1%)	28311 (30.3%) (32.9%)
E17.5 embryo (SME006)	81793	41595	30883 74.3%	28387	24656 (86.9%)	9314 (32.8%) (37.8%)

uPETs, PETs having unique sequences. Mappable ratio is the percentage of unique PETs that have one or more target locations in the genome. PET1 ratio refers to the percentage of mappable PETs that have unique targets in the genome. Pathway-asso. PET1 is the percentage of PET1 ditags that can be assigned to KEGG pathways.

### Melanin de novo biosynthesis genes are highly expressed in the cells of melanocyte origin, but not in the embryonic cells

The melanogenic potential is genetically endowed by two groups of genes encoding enzymes of Tyrosinase family and Pmel/Si family [31], which work in the up- and down-stream of the melanin biosynthesis pathway respectively. Members of the Tyrosinase family include tyrosinase precursor (encoded by *Tyr *gene), Dopachrome tautomerase precursor (*Dct*, or *Tyrp2 *gene), D-dopachrome tautomerase (*Ddt *gene), and DHICA oxidase precursor (*Tyrp1 *gene). The silver locus protein (Si) is the murine homolog of human Pmel 17 protein.

As expected, melanin biosynthesis genes were predominantly expressed in the melanocytic cells including the B16F1 melanoma cells and the Melan-a2 melanocytes, but not, or at extremely low levels, in the embryonic cells including E17.5 embryo cells and E14 embryonic stem cells (Table 2). Moreover, comparisons between melanoma cells and melanocytes revealed a pathway-wide, consistent transcriptional reduction of the major melanin biosynthesis genes in melanoma (p = 0.0018) especially for genes expressed at high level in melanocytic cells, indicating a coordinated transcriptional control for genes of the same metabolic pathway. This result thus suggests the feasibility of using PET counts for the pathway-wide quantification of gene expression levels.

**Table 2:** Expression levels of melanin biosynthesis genes.

Library Gene	B16F1 melanoma cells	Melan-a2 melanocytes	E14 Stem Cell	E17.5 embryo	Descriptions
		
	PET counts (cpm)				
*Tyr*	185	798	0	0	Tyrosinase precursor; Albino locus protein; Belong to tyrosinase gene family.
*Dct *(*Tyrp2*)	1468	3208	0	0	Dopachrome tautomerase precursor; Tyrosinase-related protein 2; Belong to tyrosinase gene family.
*Ddt*	65	58	44	0	D-dopachrome tautomerase.
*Tyrp1*	1750	2816	0	0	5,6-dihydroxyindole-2-carboxylic acid (DHICA) oxidase precursor; Belong to tyrosinase gene family.
*Si*	4751	7985	125	12	Melanocyte protein Pmel 17 precursor (Silver locus protein).

### The most significantly upregulated pathways are diversely involved in various cellular activities

A total of 7 pathways were found to be significantly upregulated (p < 0.005) in melanoma cells, and these were either related to purine, amino acid, or lipid biosynthesis, or the degradation of xenobiotic compounds (Table 3). High activation of the purine biosynthesis pathway occurred in most of the enzymes involved in ATP or GTP synthesis, starting from the biosynthesis of ribose 5-phosphate all the way through PRPP, inosinate (IMP), xanthylate, to the formation of ATP and GTP. The aminophosphonate metabolic pathway upregulation might lead to an increase in phospholipid biosynthesis. Upregulation of nitrobenzene degradation and bisphenol A degradation pathways would help melanoma cells detoxify xenobiotic compounds, in which cytochrome P_450 _plays an important role. Transcription of P_450 _*per se *was increased from an undetectable level in melanocytes to 44 cpm in melanoma cells. Biosynthesis of the amino acid tyrosine was activated in melanoma cells as suggested by the transcriptional upregulation of tyrosine biosynthesis genes. However, the utilization of tyrosine residues in melanoma cells is unclear because the transcriptional levels of the enzymes involved in consuming tyrosine for melanin synthesis in melanoma were all lower than in melanocytes (4.4, 1.6, and 2.2 fold differences for *Tyr*, *Tyrp1*, and *Dct*, respectively) and enzymes involved in downstream tyrosine utilization were mostly lower in melanoma cells. Lastly, transcriptional activation was found in most of the enzymes of the galactose pathway, especially those involved in the galactose-glucose interconversion process (Table 4) including galactosekinase 1, UDP-galactose-4-epimerase, and UDP-glucose pyrophosphorylase 2, suggesting that melanoma cells are more active than melanocytes in using galactose not only to use it as the carbon and energy sources, but also for detoxification because galactose is highly toxic if accumulated in the cell.

**Table 3:** The most significantly altered pathways in melanoma cells.

Pathway ID	Description	KEGG Category/Description*	# genes	# up-regulated genes	# down-regulated genes	p-value
up-regulated						

**230**	**Purine metabolism**	**Nucleotide Metabolism**	**119**	**11**	**23**	**4.37E-05**
**440**	**Aminophosphonate metabolism**	**Metabolism of Other Amino Acids**	**12**	**0**	**6**	**0.000153**
**626**	**Nitrobenzene degradation**	**Biodegradation of Xenobiotics**	**11**	**0**	**5**	**0.00098**
**363**	**Bisphenol A degradation**	**Biodegradation of Xenobiotics**	**16**	**0**	**6**	**0.00101**
**350**	**Tyrosine metabolism**	**Amino Acid Metabolism**	**44**	**6**	**10**	**0.00196**
**450**	**Selenoamino acid metabolism**	**Metabolism of Other Amino Acids**	**20**	**0**	**6**	**0.00370**
**52**	**Galactose metabolism**	**Carbohydrate Metabolism**	**27**	**2**	**7**	**0.00429**
4540	Gap junction	Cell Communication	85	4	14	0.00670
340	Histidine metabolism	Amino Acid Metabolism	31	1	7	0.00964
4110	Cell cycle	Growth and Death	98	2	15	0.01009

Down-regulated						

**190**	**Oxidative phosphorylation**	**Energy Metabolism**	**128**	**46**	**8**	**2.95E-27**
**193**	**ATP synthesis**	**Energy Metabolism**	**42**	**14**	**4**	**2.68E-08**
720	Reductive carboxylate cycle	Energy Metabolism	10	6	0	5.25E-06
**20**	**Citrate cycle (TCA cycle)**	**Carbohydrate Metabolism**	**26**	**9**	**2**	**6.56E-06**
**620**	**Pyruvate metabolism**	**Carbohydrate Metabolism**	**35**	**8**	**5**	**0.00057**
240	Pyrimidine metabolism	Nucleotide Metabolism	72	12	12	0.00057
660	C5-branched dibasic acid metabolism	Carbohydrate Metabolism	2	2	0	0.00319
**480**	**Glutathione metabolism**	Metabolism of Other Amino Acids/Neutralization of ROS*	**37**	**7**	**1**	**0.00400**
280	Valine, leucine and isoleucine degradation	Amino Acid Metabolism	39	7	6	0.00543

Pathways with p-values around 0.010 or lower are listed. The most significantly altered pathways (p ≤ 0.005) after being validated with manual curation are shown in bold.

**Table 4:** Expression levels of galactose-glucose interconversion pathway genes.

				PET counts (cpm)	
				
Substrates/Products	Products/Substrates	Enzyme	Gene name	Melan-a2 melanocytes	B16F1 melanoma cells
galactose	galactose 1-P	galactokinase	*Galk1*	0	154
galactose 1-P + UDP-glucose	UDP-galactose + glucose1-P	galactose 1-P uridyl transferase	*Galt*	45	33
UDP-galactose	UDP-glucose	UDP-galactose-4-epimerase	*Gale*	0	33
glucose 1-P	UDP-glucose	UDP-glucose pyrophosphorylase2	*Ugp2*	284	863

Enzymes of galactose-glucose interconversion pathway were extracted from KEGG galactose metabolism pathway.

Glycolysis is the biological process that converts glucose to pyruvate with ATP production. The process can be divided into upstream and downstream processes. The upstream process converts one molecule of glucose into one molecule of dihydroxyacetone phosphate (which is subsequently converted into glyceraldehyde 3-P) and one molecule of glyceraldehyde 3-P. The downstream process converts two glyceraldehyde 3-P molecules into two pyruvate molecules.

In melanoma cells, although the overall glycolysis pathway is not upregulated, most of the enzymes involved in the upstream reaction were upregulated, especially phosphoglucose isomerase (255:450 in cpm), which interconverts glucose 6-P and fructose 6-P, and aldolase (1263:1784 in cpm), which interconverts fructose 1,6-bisphosphate and glyceraldehydes 3-P/dihydroxyacetone phosphate.

The PI3K/Akt lipid kinase signaling pathway, which is involved in diverse cellular activities including glucose metabolism, was activated in melanoma cells. Expression levels of PI3K and Akt were 11 cpm and 176 cpm, respectively, in melanoma cells; compared with 0 cpm and 90 cpm in melanocytes.

### Most of the significantly downregulated pathways reside in mitochondria and are related to energy metabolisms through the electron transport chain

Eight pathways were found significantly downregulated (p < 0.005) in melanoma cells (Table 3). Three of them were excluded by manual curation due to the following reasons: 1) The Reductive carboxylate cycle is actually a reverse TCA cycle, and TCA cycle was already included. 2) The C5-branched dibasic acid metabolism pathway comprises only two genes, *IlvB *and *Sucla2*; and *Sucla2 *encodes a succinate-CoA ligase, which is also a component of the TCA cycle. 3) Lastly, the pyrimidine biosynthesis pathway consists of 72 gene isoforms listed in the KEGG database, of which 12 were shown to be downregulated and another 12 were shown to be upregulated (as defined by r ≤ 0.67, see Methods section), making it difficult to draw a conclusion. Thus, a total of 5 pathways were characterized as the most significantly downregulated pathways.

The top 4 of these pathways were oxidative phosphorylation (biosynthesis of ETC components), ATP synthesis (biosynthesis of ATPase subunits), TCA cycle, and pyruvate metabolism (Figure 4), all of which take place in the mitochondria and are directly or indirectly involved in ATP production through ETC. Cytochrome C (a component of oxidative phosphorylation) decreased 2.6 folds. Two of the most striking observations of the pyruvate pathway were: 1) Universal downregulation of all enzymes that convert pyruvate to Acetyl-CoA, including a 5-fold decrease for pyruvate dehydrogenase (*Pdhb*), a 6.8-fold decrease for dihydrolipoamide dehydrogenase (*Dld*), and a 2.7-fold decrease for dihydrolipoamide S-acetyltransferase (*Dlat*). 2) A10.5-fold increase of lactate dehydrogenase 1, A chain (*Ldh1*), which interconverts pyruvate and D-lactate. Thus, melanoma cells tend to consume pyruvate through anaerobic fermentation. Since Acetyl-CoA is the intermediate metabolite that enters the TCA cycle for ATP production through ETC, downregulation of Acetyl-CoA production well correlates with the downregulation of TCA cycle, oxidative phosphorylation, and ATP synthesis pathways.

**Figure 5:**
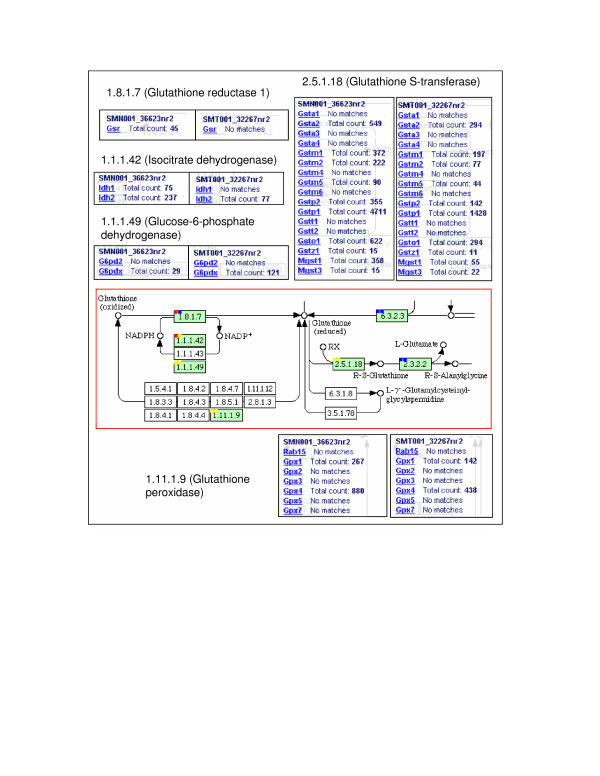
**Downregulation of glutathione metabolism in melanoma cells**. The central panel displays a partial KEGG image of the glutathione metabolism pathway. In each active 'gene box' (green), two solid squares are added to the upper left corner to reflect the associated information for the Melan-a2 melanocyte library (SMN001_36623nr2, left) and the B16F1 melanoma library (SMT001_32267nr2, right). PET counts associated with each gene isoform can be accessed by clicking on the library squares. Gene isoform-associated PET counts of the melanocyte library and the melanoma library are displayed side by side and compared to demonstrate the transcriptional downregulation of these gene isoforms in melanoma cells. PET counts are normalized to cpm (counts per million).

Glutathione molecules are the major group of non-protein thiols in the cell. They mainly act as antioxidants (e.g. Glutathione peroxidases), reducing agents (e.g. Glutathione dehydroascobate reductase), and detoxification agents (e.g. Glutathione-dependent S-transferase), and form a redox buffer in the cell through the interconversion between reduced monomers and oxidized dimers mediated by di-sulfide bond breakage or formation, respectively. Downregulation of glutathione metabolism was observed mainly in two routes: 1) the interconversion between the reduced and the oxidized forms of glutathione, and 2) the transition from the reduced glutathione to R-S-glutathione (Figure 5). In these regions, 87.5% (14/16) of the gene isoforms showed reduced expressions. Downregulation of glutathione metabolism might be related to the downregulation of the above-mentioned mitochondrial pathways. Reduced TCA cycle and ETC activities implied lower reactive oxygen species (ROS) production. Thus, the synthesis of glutathione pathway enzymes was reduced accordingly. Using proteomic and SAGE profiling de Souza et al. also observed the same trend in melanoma cell lines Tm1 and Tm5, when compared with the melan-a melanocyte from which the Tm1 and Tm5 were derived [32].

### Upregulation of RAS-MAPK pathway genes, and c-Myc in B16F1 melanoma cells

Activation of the mitogen activated protein kinase (MAPK) pathway occurs in most tumors and is a common event in uveal melanomas, although it rarely occurs through mutation of RAS or BRAF [1,33,34]. The RAS-MAPK cascade activates *c-fos, c-Myc *and stress-activated protein kinases (MSKs), which in turn phosphorylate histone H3, leading to chromatin remodeling and activation of specific genes [35].

We found transcriptional upregulation in melanoma cells across major RAS-MAPK (ERK1) pathway genes including *Ras*, *Raf*, *Mek*, and *Erk*, together with a significant isoform switch in *Ras *transcripts (Table 5). Within *Ras *isoforms, there was a 4-fold decrease for *H-ras1*, a 3-fold increase for *R-ras*, and a plausible induction of *K-ras *in melanoma cells (0 cpm in melanocytes, compared with 11 cpm in melanoma cells). Recent reports indicate that expression of the K-*ras *oncogene accelerates tumorigenesis in the context of APC deficiency [36]. In this study, PTEN, APC, and BRAF (B-type RAF kinase) transcripts were not detected in both cell types.

**Table 5:** Transcription levels of RAS-MAPK pathway genes.

	PET counts (cpm)											
	
Library	*PKA*	*PKB (Akt)*	*PKC*	*Ras*					*Raf*	*Mek1*	*Mek2*	*Erk*
								
				*H-ras*	*K-ras*	*M-ras*	*N-ras*	*R-ras*				
Melan-a2 melanocytes	0	90	15	89	0	30	15	60	58	15	44	73
								
				total: 194								

B16F1 melanoma cells	33	176	97	22	11	0	11	186	108	98	130	98
								
				total: 230								

Expression levels of the major components of RAS-MAPK pathway are listed along with that of PKA, PKB, and PKC, which are also KEGG MAPK pathway components.

The expression level of *c-Myc *was 33 cpm (with two isoforms) in melanoma cells, as compared with an undetectable level in melanocytes. c-MYC protein was shown to play a crucial role in human carcinogenesis, and β-catenin (the effector protein of the Wnt signaling) was able to activate *c-Myc *expression [37] in the presence of TCF/LEF. The enhanced expression of *c-Myc *contributes to most aspects of tumor cell biology, including cell cycle progression, cell differentiation, apoptosis, metastasis and angiogenesis [7,38]. Activated *c-Myc *[5] or *Ras *[39] can induce chromosome breakage and increase the frequency of gene amplification. Crosstalk between c-MYC and other pathways, including p53 and Wnt/β-catenin pathways has been shown to exert profound effects on many cellular aspects.

### Upregulation of Trp53, and cell cycle progression gene expressions

**Figure 6:**
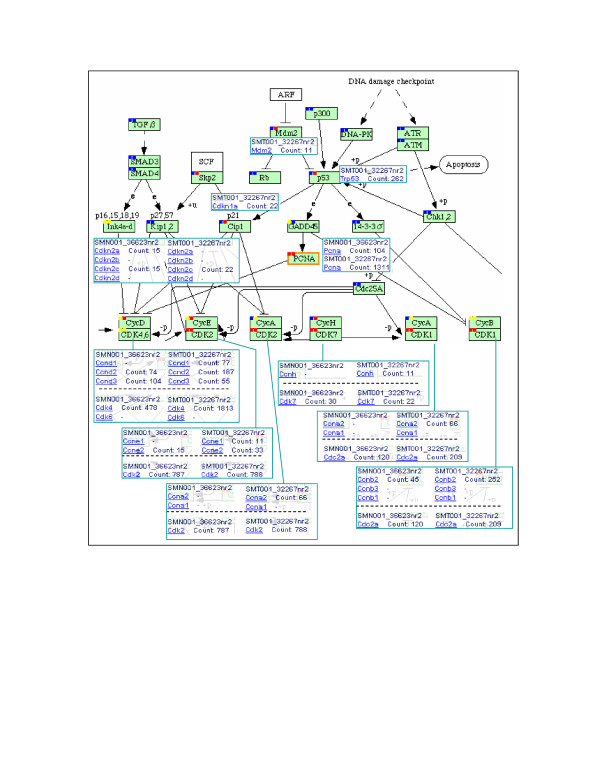
**Upregulation of cyclins and CDKs in melanoma cells**. Part of KEGG cell cycle pathway is shown on top with a focus on cyclins and CDKs and their associated PET counts. Two solid squares are added to each active 'gene box' (green) for accessing PET data related to gene isoforms: the left square links to the Melan-a2 melanocyte library (SMN001_36623nr2), while the right square links to the B16F1 melanoma library (SMT001_32267nr2). The degree of PET-to-gene isoform matching is indicated by different colors: blue, no match at all; yellow, partially matched; and red, all isoforms matched (by PETs). Boxes in the lower portion show PET counts for the gene isoforms of each pair of cyclin (top) and CDK (bottom). PET counts have been normalized to cpm (counts per million).

p53 plays an important role in suppressing tumor development and p53 mutations have been found in 50% of human cancer patients [40]. The mouse p53-encoding gene *Trp53 *of B16F1 was previously characterized as a wild-type by genetic studies [13] and the p53 protein, although expressed at low level, was localized to the nucleus and thus expected to be functionally normal. However, we detected a level of 262 cpm for *Trp53 *mRNA in B16F1 melanoma cells, in contrast to an undetectable level in Melan-a2 melanocytes (Figure 6). The upward transcriptional regulation of p53 did not seem to be compatible with its (low) protein level and might imply a translational control or protein degradation of p53 in melanoma cells. Genetic studies also identified a deletion spanning across the overlapping loci encoding p16^INK4a ^and p19^ARF ^proteins (mouse *Cdkn2a *of Ink4a-d, shown on the left-hand side of Figure 6) [13,41]. The deletion is supposed to derepress the cell cycle arrest by INK4a protein, which would otherwise binds to CDK4,6 and causes cell cycle arrest at G1 phase. In line with the report, the *Cdkn2a *(and *Arf*) transcript was not detected in melanoma cells. ARF protein counteracts the degradation of p53 by MDM2; thus, deletion in the *Arf *gene would reduce the protein level of p53. It is likely that the transcription activation of p53 in melanoma cells was due to activation by c-MYC. The presence of the E-box in the p53 promoter directly places p53 under the transcriptional control by c-MYC, and it is not surprising to see co-activation of p53 and c-MYC [42,43]. Elevation of p53 pathway expression was also shown in cMYC induced DNA damage and RAS activation [44].

Besides p53, the tumor suppressor genes *Atm *and *Cdkn1a *(p21 gene) were expressed higher in melanoma cells than melanocytes (65:0 and 22:0 in cpm, respectively). It was reported that the expression levels of c-MYC, proliferating cell nuclear antigen (PCNA), and p53 were all higher in metastasizing colorectal cancer than non-metastasizing tumors as detected by immunohistochemistry [6].

Cell cycle progression is tightly regulated by two types of controls: a rhythmic expression of cyclins in conjunction with their interactions with their kinase partners, and a supervisory control through diversified checkpoint monitoring mechanisms [45]. PCNA, a nuclear protein used as an indicator of cell proliferation, increased from 104 cpm in melanocyte to 1311 cpm in melanoma (12.6 fold), implying aggressive growth of melanoma cells. In general, compared to melanocytes, the transcriptional levels of cyclins and cyclin-dependent kinases are all elevated in melanoma cells (Figure 6).

### Perturbations in apoptosis pathway: elevation of caspase 8 transcription and E2f1-to-E2f4 switch

Two alterations were apparent in melanoma apoptosis pathway: elevation of caspase 8 transcription and an *E2f1*-to-*E2f4 *gene isoform switch.

Expression of caspase 8 was significantly increased (0:154 cpm) in melanoma cells. Both *Bad *and *Bax *expression levels were increased slightly and *Bad *showed isoform induction, suggesting that there might be apoptotic pressures, causing the pathway to be upregulated at some points. However, like the p53 pathway, the apoptosis pathway seemed to be disoriented and deregulated.

We found an *E2f1*-to-*E2f4 *switch in melanoma cells, wherein p73 transcript was not present. E2Fs are known to be crucial regulators of a variety of cellular events including cell cycle progression, DNA replication, checkpoint control, apoptosis, and DNA repair [46], and E2F1 is crucial for E2F-dependent apoptosis [47,48]. In general, E2Fs can be divided into activators (E2F1-3) and repressors (E2F4 and E2F5). However, it is technically possible for the repressors to activate certain genes. E2F1 induces the transcription of p73 which in turn activates the apoptosis pathway [40,49]. The capability of p73 to induce apoptosis in *TP53*^-/- ^cells is a p53-independent tumor control mechanism that runs in parallel with p53-dependent apoptosis. There were six *E2f *gene isoforms (*E2f1*-*E2f6*) listed in KEGG database, among which melanocytes expressed *E2f1 *(60 cpm) and *E2f5 *(15 cpm), while melanoma cells expressed *E2f4 *(11 cpm) and *E2f5 *(11 cpm). It seems likely that melanoma cells adopt the *E2f1*-to-*E2f4 *switch as a strategy to avoid E2F1-p73-dependent apoptosis. As E2Fs regulate a broad spectrum of cellular activities, it remains to be learned how such a switch in *E2f *isoforms affects overall cancer cell physiology. It is possible that melanoma cells may still express *E2f1 *at a redefined basal level below our detection limit to sustain the transcription of certain genes.

### Cell adhesion and metastasis related genes were expressed as diverse isoforms in melanoma cells

Recently β-catenin has drawn a great attention due to its dual role in signal transduction and cell adhesion. It is not only a transcription factor that activates genes such as *c-Myc*, but also a structural adaptor protein that bridges extracellular cadherins through α-catenin to the intracellular actin cytoskeleton network, which is crucial for cell-cell adhesion and migration [9].

Metastases are responsible for most cancer deaths and the involvement of adhesion proteins in metastasis has been well documented [1,8]. Since B16F1 is a metastatic melanoma cell line, it is of particular interest to find out what adhesion proteins are expressed in melanoma cells. In both normal melanocytes and melanoma cells, transcripts of extracellular adhesion proteins such as nectin (weak cell-cell glue protein) and cadherin (strong cell-cell glue protein) were low or not detected (Table 6), suggesting that minimal cell-cell contact through cadherin was a common scheme for both cell lines. However, it could be an artifact resulted from the process of cell culture. Like that of β-catenin, integrin expression was very diverse in melanoma cells. The two expressed integrin genes included: full-length (exon 1–16) *Itgb1 *(expressed in both cell types, but melanoma cells had one extra isoform with the PAS located in exon 10); and *Itgb5 *(specifically expressed 3 different types of isoforms – with different TSSs – in melanocytes). An 8-fold increase for CD44 isoforms (S1E14T14, accompanied with S1E12T14 and S1E4T14 that were expressed at lower level) was found in melanoma cells. Since CD44 is able to induce integrin-mediated adhesion of colon cancer cell lines to endothelial cells [50], the process by which CD44 interacts with such diversified integrin isoforms is an interesting issue to be addressed. Moreover, CD44 was identified as a marker of cancer progression and its variants play a role in metastasis [51]. There were 4.6× and 5.8× increases for *Actb *and *Actg1 *isoforms of the cytoskeleton protein actin, respectively, in melanoma cells as compared with melanocytes. The increase in actin expression may not be relevant to metastasis; it may contribute to the identity of melanoma skin cancers as solid tumors instead.

**Table 6:** Transcription levels of TSS/PAS variants of cell adhesion-related proteins.

		PET counts (cpm)		A:B ratio
		
Protein	Gene isoforms	Melan-a2 melanocytes (A)	B16F1 melanoma cells (B)	
Collagen	*Col2a1*	*S6-7E53T53: 15	0	30:0
	*Col1a2*	S31E52T52: 15	0	
Integrin	*Itgb1*	S1E16T16: 44	S1E16T16: 11 S1E10T16: 11	44:22
	*Itgb5*	S1E16T16: 15 *S6-7E16T16: 15 S14E16T16: 15	0	45:0
Actin	*Actb*	S1E2T6: 15 S1E6T6: 399	S4E6T6: 11 S1E2T6: 1896	414:1907
	*Actg1*	S3E6T6: 15 S4E6T6: 15 S1E6T6: 876	S1E6T6: 5260	906:5260
Cadherin	*Cdh11 *(not in KEGG)	*S2-3E6-7T13: 15 *S3E6-7T13: 15	*S2-3E6-7T13: 11	30:11
JAM	*Jam2*	S1E10T10: 15	0	15:0
	*Jam3*	S1E9T9: 15	0	15:0
β-catenin	*Catnb*	S1E1T15: 30 (skip*) S1E15T15: 15	S11E15T15: 33	15:33
α-catenin	*Catna1*	S4E18T18: 15	S1E18T18: 22 S1E5T18: 11 S15E18T18: 11	15:44
Snail, Slug	*Snail*, *Slug*	0	0	0:0
CD44	*Cd44*	S1E14T14: 15	S1E14T14: 77 S1E12T14: 33 S1E4T14: 11	l5:121
OPN (osteopontin)	*Spp1*	S1E7T7: 1889	0	1889:0

'S', starting exon; 'E', ending-exon; 'T', total number of exons. *Hyphen (-), matching to intron region.

### TSS/PAS variants

Besides the transcript variants resulted from alternative splicing, transcriptions using alternative TSSs or PASs generate different types of transcripts with variations in the 5'UTRs or 3'UTRs, which are more likely to be involved in regulation of gene expression compared with other (internal) exons. One of the unique advantages of PET approach is its capability to precisely map the 5' and 3' boundaries specified by alternative TSSs and alternative PASs, respectively. It was reported that a total of 5,401 genes were found to be alternatively spliced in Melan-c and B16-F10Y melanoma cell lines [25]. Melan-a2 melanocytes and B16F1 melanoma cells are similar to Melan-c melanocyte and B16-F10Y melanoma cells, respectively. Among the total of 5,606 genes identified from melanoma and melanocyte cells, 1,289 genes (23.0%) exhibited alternative TSSs/PASs, 2,714 genes (48.4%) showed library-specific expressions, and 1,603 genes (28.6%) expressed only common transcripts (Table 7). In theory, the percentage of genes using alternative TSSs/PASs is more likely to increase as the library size increases. Transcriptional control with alternative TSSs or PASs seems to be a common mechanism for melanocytic cells.

**Table 7:** Differential gene expression between B16F1 melanoma cells and Melan-a2 melanocytes.

Category	1 (A)	2 (B)	3 (C)	4 (AB)	5 (AC)	6 (BC)	7 (ABC)	Total genes	Total transcripts
#Gene	1485	1229	1603	258	437	374	220	5606	
#Melan-a2 melanocyte-specific tsc (A)	1678	0	0	299	549	0	351		2877
#B16F1 melanoma-specific tsc (B)	0	1409	0	323	0	473	382		2587
#Overlapped tsc (C)	0	0	1681	0	485	423	317		2906
									8370

Known genes expressed in Melan-a2 melanocytes and B16F1 melanoma cells are split into 7 categories based on the combination of their transcript types. Transcripts can be Melan-a2-specific (A), B16F1-specific (B), or common to both (C); the 7 categories represent all possible combinations. For example, each gene in category 1 expresses one or more transcript solely in melanocytes; each gene in category 4 expresses in both cell types but has no cross-library overlap in transcript type; and so on. 'tsc', transcript(s).

### Transcription of solute carriers (SLCs)

To gain insight into perturbations in mitochondria, we analyzed the expression of a group of membrane transporter proteins called solute carriers (SLCs) [52]. A total of 67 SLCs were detected in melanoma and/or melanocyte transcriptomes. To increase the accuracy of the data, we excluded the PETs of less than 20 cpm because these were single count PETs before normalization, resulting in 44 SLCs of reasonable confidence (Table 8). Four discoveries were made from this dataset: i) SLCs heavily expressed in melanocytes remained heavily expressed in melanoma, especially Slc25a5, Slc3a2, and Slc25a3. ii) Among these highly expressed SLCs, Slc25a5 (an ATP/ADP transporter) increased by 3.2 folds and was the most noticeable perturbation in SLC expression. iii) SLCs specific to melanoma cells or melanocytes were expressed at a much lower level, normally less than 100 cpm, except Slc6a15 (amino acid and osmolyte transporter) which had a cpm of 109. iv) As shown by the cell type specific SLCs, melanoma cells seemed to be more active in the transportation of amino acids (e.g. Slc6a15, Slc25a13, and Slc7a7), fatty acids (e.g. Slc27a4), UDP-galactose (e.g. Slc35a2, Slc35a4), Mg^++ ^ion (e.g. Slc41a3), and citrate/malate (e.g. Slc25a10 [53]). The melanoma-specific SLC expression levels and Slc25a5 activation level correlated well with the activations of the purine, galactose, and amino acid related metabolic pathways. When these pathways were activated, production of the proteins involved in the transportation of their substrate and/or products was also increased accordingly.

**Table 8:** Transcriptional alterations of solute carriers (SLCs).

SLCs	Solute(s)	#A-s tsc	A-s cpm	#A/B -c tsc	A-c cpm	B-c cpm	#B-s tsc	B-s cpm	(A-s + A-c) cpm	(B-s + B-c) cpm	B – A cpm
Slc25a5	adenine nucleotide (ATP/ADP)	1	15	1	842	2631	3	109	857	2740	1883
Slc3a2	neutral amino acids	2	44	2	1553	1305	5	152	1597	1457	-140
Slc25a3	phosphate	0	0	2	1423	1174	1	43	1423	1217	-206
Slc25a11	2-oxoglutarate/malate	1	15	1	131	120	2	33	146	153	7
Slc20a2	phosphate	1	15	1	189	120	2	22	204	142	-62
Slc2a1	glucose	1	15	1	15	120	0	0	30	120	90
Slc31a1	copper	1	15	1	58	98	0	0	73	98	25
Slc30a9	zinc	1	15	1	29	65	0	0	44	65	21
Slc12a4	K+/Cl-	1	15	1	44	54	0	0	59	54	-5
Slc35a2	UDP-galactose	1	15	0	0	0	2	54	15	54	39
Slc25a17	adenine nucleotide	1	15	1	116	33	0	0	131	33	-98
Slc25a19	adenine nucleotide	2	29	1	44	33	0	0	73	33	-40
Slc9a3r1	Na+/H+ exchanger	2	29	1	116	11	1	11	145	22	-123
Slc6a9	neurotransmitter, glycine	0	0	1	15	22	0	0	15	22	7
Slc35c2	neutral amino acids	0	0	1	58	11	0	0	58	11	-47
Slc39a6	zinc	1	15	1	15	11	0	0	30	11	-19
											
Slc6a15	neurotransmitter, aa, osmolytes	0	0	0	0	0	1	109	0	109	109
Slc25a13	aa Q and E	0	0	0	0	0	3	76	0	76	76
Slc2a8	glucose	0	0	0	0	0	1	54	0	54	54
Slco3a1	organic anion	0	0	0	0	0	1	43	0	43	43
Slc35a4	UDP-galactose	0	0	0	0	0	1	33	0	33	33
Slc41a3	mg++	0	0	0	0	0	1	33	0	33	33
Slc12a5	Na+/Cl-	0	0	0	0	0	1	22	0	22	22
Slc25a10	citrate/malate, dicarboxylate	0	0	0	0	0	1	22	0	22	22
Slc27a4	fatty acids	0	0	0	0	0	1	22	0	22	22
Slc37a3	glycerol-3-P	0	0	0	0	0	1	22	0	22	22
Slc37a4	glycerol-6-P	0	0	0	0	0	1	22	0	22	22
Slc4a2	anion	0	0	0	0	0	1	22	0	22	22
Slc7a7	cation amino acid	0	0	0	0	0	1	22	0	22	22
											
Slc35a1	CMP-Sialic acid	2	87	0	0	0	0	0	87	0	-87
Slc37a2	glycerol-3-P	4	73	0	0	0	0	0	73	0	-73
Slc15a4	peptide	1	58	0	0	0	0	0	58	0	-58
Slc25a1	citrate	2	58	0	0	0	0	0	58	0	-58
Slc37a1	glycerol-3-P	1	44	0	0	0	0	0	44	0	-44
Slc10a3	Na+/bile acid	2	29	0	0	0	0	0	29	0	-29
Slc22a18	organic cation	1	29	0	0	0	0	0	29	0	-29
Slc33a1	acetyl-CoA	2	29	0	0	0	0	0	29	0	-29
Slc39a1	zinc	1	29	0	0	0	0	0	29	0	-29
Slc39a11	metal ion	1	29	0	0	0	0	0	29	0	-29
Slc39a3	zinc	1	29	0	0	0	0	0	29	0	-29
Slc4a8	anion	1	29	0	0	0	0	0	29	0	-29
Slc6a6	taurine	2	29	0	0	0	0	0	29	0	-29
Slco4a1	anion	1	29	0	0	0	0	0	29	0	-29

'A', Melan-a2 melanocytes; 'B', B16F1 melanoma cells; A/B, for both A and B; '#', number of transcripts; 's', specific; 'c', common; 'tsc', transcript; 'cpm', PET counts per million; upper panel, common; central panel, melanoma-specific; lower panel, melanocyte-specific; SLCs with PET counts less than 20 cpm for both libraries were excluded.

## Conclusion

This study aimed to reveal global pathway aberrations in melanoma cells using the robust GIS-PET technology in conjunction with KEGG pathway database and hypergeometric distribution analysis. Surprisingly, all the most significantly altered pathways, including 7 upregulated and 5 downregulated pathways, are metabolic pathways. The most significantly upregulated melanoma pathways are very diverse as they are involved in a broad spectrum of cellular activities including purine and amino acid biosyntheses, galactose utilization, and detoxification of various harmful compounds. On the other hand, the most significantly downregulated pathways are tightly correlated, making the conclusion highly reliable and trustworthy. Here, for the first time in melanoma, we provide compelling evidence to show that cancer cells tend to avoid using the most efficient route of ATP production through electron transport chain, which is located in mitochondrial inner membrane and which uses oxygen as the electron receptor.

This notion is mainly based on the observation that mitochondria-harbored metabolic pathways are severely downregulated. In eukaryotic cells, the top 4 of the most significantly downregulated melanoma pathways constitute an important catabolic flow for intermediate metabolite and energy productions (from pyruvate metabolism, through TCA cycle and oxidative phosphorylation, to ATP synthesis). To further avoid using this route, melanoma cells tend to consume pyruvate molecules to produce D-lactate instead of Acetyl-CoA (a substrate for TCA cycle). Moreover, in parallel with the metabolic alterations, mitochondrial permeability and transportation system are also redefined. Among the SLCs, we detected upregulation of citrate/malate exchanger Slc25a10. In exchange for malate from cytosol, the citrate/malate exchanger exports citrate from mitochondria into cytosol for lipid biosynthesis [53]. Since citrate is an intermediate metabolite of TCA cycle for the generations of NADH and FADH_2_, which in turn are passed on to the respiratory chain for ATP production, elevation of the citrate/malate exchanger is an additional step to reduce the capacity for ATP production through the ETC.

Our data also indicated that melanoma cells increased the consumption of glucose and galactose for ATP synthesis and intermediate metabolite production. In melanoma cells, galactose pathway and the galactose-glucose conversion pathway were both transcriptionally upregulated. We suspect that these upregulations aimed not only to support cancer cell proliferation, but also to reduce the intracellular concentration of galactose, which is a potent toxin for the cell. Running side by side with the upregulated detoxification pathways, transcriptional upregulation of the galactose-glucose interconversion pathway would neutralize the galactose toxicity, which can be critical in the solid tumor environment where circulation is much more limited than normal cells. Notice that the expression of Slc35a2, an UDP-galactose transporter, was also upregulated by 3.8 folds in melanoma cells. The increased consumption of carbohydrates, intermediate metabolites and other nutrients from the circulation system for tumor growth eventually leads to cancer cachexia [54].

It is interesting to compare our results with that of Vahsen et al [55]. AIF (apoptosis inducing factor) is a protein that, in response to apoptosis induction, translocates from mitochondria to the nucleus to exert its chromatinolytic activity. They described that human or mouse cells lacking AIF would exhibit high lactate production and enhanced dependency on glycolytic ATP production due to severe reduction of ETC complex I activity. Moreover, mice with reduced AIF expression demonstrated a reduced oxidative phosphorylation. Their results suggested a role of AIF in the biogenesis and/or maintenance of complex I of the electron transport chain. In our investigation, AIF transcript was not detected in both melanoma and melanocyte cells and thus did not contribute to the physiological differences between melanoma cells and melanocytes. However, the expression levels of the ETC pathway as well as the above-mentioned metabolic pathways were all lower in melanoma cells. Thus, the same phenomena were observed with different approaches.

Then, how is the slowdown of mitochondrial activities related to cancer cell survival? Due to the critical role of mitochondria in apoptosis [56] and the importance of angiogenesis in cancer neoplasia, we argue that downregulation of mitochondrial pathways leading to the reduction of ETC usage by melanoma cells is actually a "one stone for two birds" strategy, aiming to downgrade the role of mitochondria in apoptosis and to reduce the dependency of the cell on angiogenesis for oxygen supply (and toxic material removal), which is very limited during the early stage of angiogenesis. Thus, these issues are tightly correlated and melanoma cells sacrifice the most efficient way of ATP production in exchange, at least, for a reduced threat from apoptosis and a reduced dependency on angiogenesis. This notion further emphasizes the importance of mitochondria for normal cellular functions and deregulated mitochondria would favor the survival of tumor cells. Notice that enhanced detoxification capability would also reduce the dependency on angiogenesis for the removal of toxic compounds.

It is interesting to learn how melanoma cells can still manage to grow vigorously (as indicated by the 12.6-fold increase of PCNA expression level) despite the surveillance of cell cycle progression checkpoints and apoptotic pathways in the context of such drastic alterations in multiple pathways. Based on the coexistence of the transcriptional upregulation of *Trp53 *and *Ink4a*/*Arf *deletion in the p53 pathway, the deregulated cell cycle progression, and the scattered pattern of alterations in the apoptosis pathways, etc., both tumor suppressor mechanism and apoptosis pathway seemed to be shattered into discrete pieces in which the coordination between the components no longer existed. How these pathways are deregulated and how changes at the p53 transcription level are translated to changes in the protein and protein phosphorylation, which is required for p53 activation, remains to be understood.

Based on the notion that cancers generally develop from multiple mutations in diverse genes, we would expect multiple pathways to be altered, as has been shown in our data. Due to the extensiveness of the renovation of melanoma pathways, the selectivity for certain anabolic, catabolic, or signal transduction pathways to be upregulated or downregulated for melanoma cell survival seem to be taking place through a series of micro-evolution process involving numerous testing of mutations and selections in the context of carcinogenesis environment inside the tumor mass.

Although pathway studies reflect the combinatorial outcomes of functionally related genes and are supposed to be more reliable than studies of individual genes or gene sets, the results, however, were generated only from B16F1 melanoma cell line. The generality need to be confirmed with other cancer cell lines or cancer tissue samples. By such additional works, we should also be able to further clarify how many of these changes were resulted from adaptation of the cells to *in vitro *culture conditions.

Moreover, the hypergeometric distribution approach can be made even more powerful through improving the resolution by looking into the sub-pathways for each pathway. As described in the study of the pyruvate metabolic pathway, a sub-pathway may be favored against the others. Under this situation, the resolution of hypergeometric distribution would be compromised. To address this issue, one would have to dissect each pathway into distinct sub-pathways, which requires laborious functional studies on each pathway and is a component of our future work.

In summary, by using the GIS-PET technology in combination with KEGG database and hypergeometric distribution analysis, we demonstrate the most significantly upregulated and downregulated melanoma pathways and reemphasize the importance of mitochondrial deregulation for cancer survival. Enzymes of the altered pathways, such as the galactose-glucose interconversion pathway, signaling pathway components that have been altered, and the upregulated transporter proteins in melanoma cells can be tested as drug targets for cancer therapy. We believe this approach, which can be readily correlated with other PET approaches such as ChIP-PET, will significantly facilitate our understanding of gene expression and regulation at the pathway level.

## Abbreviations

CSA Compressed suffix array

ETC Electron transport chain, or respiratory chain

KEGG Kyoto Encyclopedia of Genes and Genome

PAS Polyadenylation site

PCR Polymerase chain reaction

PET Paired-End diTag, indicating either the technology or the PET sequence generated from the technology

PET PET technology or PET ditag (sequence)

ROS Reactive oxygen species

TCA cycle Tricarboxylic acid cycle, or citric acid cycle, or Krebs cycle

TF Transcription factor

TFBS Transcription factor binding site

TSS Transcription start site

UTR Untranslated region

## Competing interests

The author(s) declare that they have no competing interests.

## Authors' contributions

KPC conceptualized the approach of using PET transcriptome data in conjunction with KEGG database for pathway and library comparisons, and analyzed data. PA setup the local KEGG pathway database, and coded programs for pathway analysis. HX conducted statistical analyses. AT coded programs for data analysis and visualization. PN constructed libraries. RY and CLW contributed to the PET method development. ETBL provided some ideas for data analysis. CLW also involved in manuscript preparation. WKKS developed the CSA approach for PET mapping. KPC wrote the paper. All authors have read and approved the final manuscript.

## Pre-publication history

The pre-publication history for this paper can be accessed here:


